# Activity Dependence of a Novel Lectin Family on Structure and Carbohydrate-Binding Properties

**DOI:** 10.3390/molecules25010150

**Published:** 2019-12-30

**Authors:** Irina Chikalovets, Alina Filshtein, Valentina Molchanova, Tatyana Mizgina, Pavel Lukyanov, Olga Nedashkovskaya, Kuo-Feng Hua, Oleg Chernikov

**Affiliations:** 1 G.B. Elyakov Pacific Institute of Bioorganic Chemistry, Far Eastern Branch of the Russian Academy of Sciences, Vladivostok 690022, Russia; 2 School of Natural Sciences, Far Eastern Federal University, Vladivostok 690950, Russia; 3 Department of Biotechnology and Animal Science, National Ilan University, Ilan 260, Taiwan; 4 Department of Pathology, Tri-Service General Hospital, National Defense Medical Center, Taipei 114, Taiwan

**Keywords:** lectin, mussel, Gal-specific, mytilectin family, carbohydrate specificity, *Crenomytilus grayanus*, *Mytilus trossulus*

## Abstract

A GalNAc/Gal-specific lectins named CGL and MTL were isolated and characterized from the edible mussels *Crenomytilus grayanus* and *Mytilus trossulus*. Amino acid sequence analysis of these lectins showed that they, together with another lectin MytiLec-1, formed a novel lectin family, adopting β-trefoil fold. In this mini review we discuss the structure, oligomerization, and carbohydrate-binding properties of a novel lectin family. We describe also the antibacterial, antifungal, and antiproliferative activities of these lectins and report about dependence of activities on molecular properties. Summarizing, CGL, MTL, and MytiLec-1 could be involved in the immunity in mollusks and may become a basis for the elaboration of new diagnostic tools or treatments for a variety of cancers.

## 1. Introduction

Lectins are known as carbohydrate-binding proteins which can be found in different kinds of organisms from viruses to mammals. They take part in interaction between cells, cell and matrix, and organisms. These proteins are able to bind the whole carbohydrate molecule, its part, or even the glycosidic linkage [1]. In addition to recognizing pathogens, lectins also participate in other types of biological processes, such as intercellular interaction, protein transport and synthesis, and signal transduction. [2]. Due to the capability of distinguishing sugar structures, lectins are used not only as useful biochemical reagents in many research fields, including glycomics, but they are promising compounds for biomedical application [3]. Recently, interest in lectin drug potential has increased significantly, especially as a cancer treatment [4], as well as adjuvants or modulators of immune responses [5] and antiviral agents [6].

The existing data mainly concerns vertebrates lectins, and much less information is obtained about lectins isolated from invertebrates, especially from non-model organisms. The functional mechanisms of invertebrate lectins for the recognition of carbohydrate ligands are still poorly studied, not only because of insufficient work, but also because of the lack of homology with well-studied vertebrate lectins and the large number and diversity of lectins from different invertebrate organisms. The ubiquitous presence of lectins in invertebrates assumes a phylogenetic distribution and indicates various specificities. Practically all classes and subclasses of invertebrates examined have lectins. These include crabs, snails, worms, insects, mollusks, and sponges [1]. Lectins are present mainly in hemolymph and gonads, as well as on the membranes of hemocytes, key cells of innate immunity. [7]. The invertebrate humoral lectins have been proposed to act as opsonins, hemolysin, and sugar specific antibodies like molecules [8].

Today a large number of galactosyl-binding lectins have been reported to occur in the invertebrates, which bind to D-galactose, its derivatives, and D-galactose containing complex carbohydrates in a very selective manner [9,10,11,12,13].

## 2. Novel Lectin Family Structure

**Figure 1:**
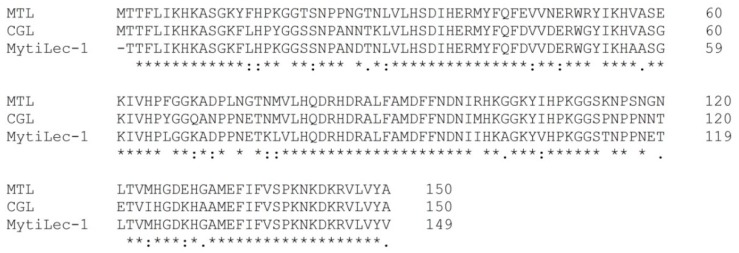
Alignment of MTL (AKI29293.1), CGL (AEY80387.1) and MytiLec-1 (B3EWR1) generated by Clustal Omega (https://www.ebi.ac.uk/Tools/msa/clustalo/).

New Gal-specific lectin MytiLec (later renamed to MytiLec-1 [14], the new name was used in present review) with globotriose-dependent cytotoxicity was recently identified in the mussel *Mytilus galloprovincialis* [12], and later it was reported about lectin CGL from the mussel *Crenomytilus grayanus* with similar amino acid sequence and antibacterial activity [15]. Analysis of the protein sequences of these lectins (Figure 1) showed that they form a novel lectin family, sharing common structure—three tandem repeats with similar sequences to each other consisting of 40 amino acids and three carbohydrate-binding sites in each sub-domain. According to classification of the protein based on domain identification using the protein family databases Pfam [16] and InterPro (www.ebi.ac.uk/interpro/), all MytiLec-like sequences belong to the β-trefoil fold superfamily [17]. The 3-D structures of MytiLec-1 and CGL involve a β-trefoil fold, which is also common for R-type lectins, a widespread lectin family found in almost all taxa [18,19]. Due to a significant degree of similarity a novel structural lectin family which contains all the MytiLec-like sequences found in bivalves can be suggested. This view was fully confirmed by a transcriptome analysis performed in *M. galloprovincialis*, which indicated the presence in the same species of two other sequences named MytiLec-2 and MytiLec-3 similar to MytiLec-1 [20]. A new GalNAc/Gal-specific lectin (MTL) from sea mussel *Mytilus trossulus* was later isolated and characterized [21]. The genera Mytilus and Crenomytilus are close to each other according genome analysis [22]. A BLAST (Basic Local Alignment Search Tool) search revealed high identity and similarity of MTL with CGL and MytiLec-1 and a complete lack of homology with other known lectins. According to PHYRE2 (Protein Homology/analogY Recognition Engine V 2.0) data, MTL, MytiLec-1, and CGL share a common β-trefoil fold [11]. CGL, MTL, and MytiLec-1 are similar to each other in regard to the basic physicochemical properties (Table 1) and carbohydrate specificity (Table 2).

Lectins possess several specific activities against the cells: blood group dependent hemagglutination, agglutination of tumor cells, and mitogenic activation of lymphocytes. The binding of lectins to the carbohydrate components of cell membranes initiates a wide range of molecular processes inside the cell. The affinity of individual carbohydrate-binding sites is rather weak, and high glycan binding activity is achieved through interaction with several sites. As we already mentioned, CGL, MTL, and MytiLec-1 share a tertiary structure containing β-trefoil fold. Structures like that can form oligomers due to the internal symmetry. Multivalency and oligomerization are significant for carbohydrate-binding proteins, such as lectins [23].

MytiLec-1 and CGL were originally reported to be monomers [10,12,15]. Recently, the crystal structures of MytiLec-1 and CGL were determined [18,19]. An important conclusion from the analysis of CGL structure is that the lectin can contain up to a total of six ligand-binding sites owing to dimeric quaternary structure and the triplication of the ligand-binding site in each protomer.

It confirms the results about an oligomerization of CGL due to hydrophobic interaction. Formation of partially insoluble precipitate at high concentrations or during prolonged storage of CGL was detected. The minimum CGL concentration at which protein self-association began was defined by mass spectroscopy. The dimer formation was observed at a concentration of 0.01 mg/mL, and at 0.1 mg/mL an insignificant amount of the tetramer appeared [24].

Protein oligomerization during storage was observed for galectins and galactoside-binding lectins [25]. It has been shown that oligomerization of CGL due to collagen-like domain is significant for its biological properties. It should be noted the functional importance of the interaction of lectin in vivo with collagenase (or gelatinase, which is present in the tissues or intracellular components). Degradation of the collagen-like domain can reduce the agglutinating activity of lectin and change its carbohydrate-binding properties [24]. Thus, proteolysis is able to regulate the function of lectin in vivo.

MytiLec-1 is also dimer in its native state, according data obtained by analytical ultracentrifugation [18]. The two monomers in the asymmetric unit are closely associated by hydrophobic interactions and hydrogen bonds. This may imply polyvalence, which is crucial for some biological functions such as hemagglutination or interaction with glycans of a cellular surface. Monomeric mutant MytiLec-F93DF94S had a weak hemagglutinating activity, about 10 times less than that of the dimer [18]. Mitsuba-1, the monomeric form created by symmetry constraining the structure of a MytiLec-1 subunit, showed no hemagglutinating activity at any concentration tested [26]. Despite the loss of hemagglutinating activity CGL and MytiLec-1 showed glycan binding activity. Mitsuba-1 failed to agglutinate Raji cells, but it was observed to bind them [26]. The interaction of CGL-treated collagenase with enzyme-linked BSM was studied by enzyme-linked immunosorbent assay. The results show that the binding of this fragment to BSM was almost 10% more than of native CGL, which confirmed the lectin carbohydrate-binding site was not only unaffected during the cleavage but also rendered more available to BSM, a glycoprotein containing carbohydrate chains for which the lectin was highly specific [24].

## 3. Glycan Specificity

After analyzing the CGL crystal structure [19], it was found that lectin binds to galactose in different ways: CGL carbohydrate-binding sites are exposed to solvent and include a shallow cleft that can contain both α- and β-anomers of galactose. In the crystal structure, CGL binds galactose as an α-anomer, with the exception of Site 3, which recognizes β-anomer, whereas in the complex of MytiLec-1 with GalNAc was not observed significant occupancy by β-anomer at any site according a difference electron density map. It would be interesting to investigate binding with ligands of MTL whose crystal structure is still not received.

To understand more about detailed carbohydrate recognition ability we submitted CGL and MTL samples to the Consortium for the Functional Glycomics (CFG) for glycan array analysis (http://www.functionalglycomics.org/). The array consists of natural and synthetic mammalian glycans with amino linkers printed onto glass slides. Microarray format was used to evaluate the binding properties of CGL and MTL. Results on the binding with all glycans are accessed through the Functional Glycomics Gateway. The glycans with high affinity are listed in Table 3.

The obvious binding affinity for weaker ligands can be influenced by ligand density and protein concentration [28], but the rank order is relatively stable. In other words, the compounds can be grouped into two subsets, with α- or β-anomers of galactose at their non-reducing ends, and the top five compounds present the examples of each of these subsets. The establishment of the glycan binding profile showed that CGL and MTL are capable of binding both α- and β-galactose, but the binding of CGL to glycans with terminal α-Gal is stronger. It appears that CGL possessed slight affinity to β-Gal in highly branched glycans only. On the contrary MTL binds branched β-Gal-terminated glycans stronger and shows weak affinity for unbranched glycans with α-Gal on the end of chains (Table 3).

These data were confirmed using the GlycanMotifMiner tool on the GlycoPattern website (https://glycopattern.emory.edu) to detect motifs in the glycan array. Analysis of the most common glycan motifs revealed two main patterns with high affinity: Galα1-4Galβ1-4GlcNAc for CGL and Galβ1-4GlcNAcβ1-3Galβ1-4GlcNAcβ1-3Galβ1-4GlcNAcβ1-2Man for MTL. Perhaps, such difference in preferences can affect biological properties of these lectins.

## 4. Effect on Bacteria and Fungi

Marine organisms live in direct contact with the environment, surrounded by high concentrations of pathogenic viruses, bacteria, and fungi. Innate immunity is the first line of protection against disease [29]. Lectins that agglutinate pathogens act as pattern recognition receptors (PRRs), the binding of which directly to microorganisms may depend on the recognition of molecules of the microbial cell wall, especially the carbohydrate groups [20]. PRRs include evolutionarily conserved extracellular, membrane-bound, and cytosolic molecules that play an essential role in the primal part of the immune system recognizing the pathogen-associated molecular patterns (PAMPs) [30] and are very important for invertebrate organisms that lack an adaptive immune system.

**Figure 2:**
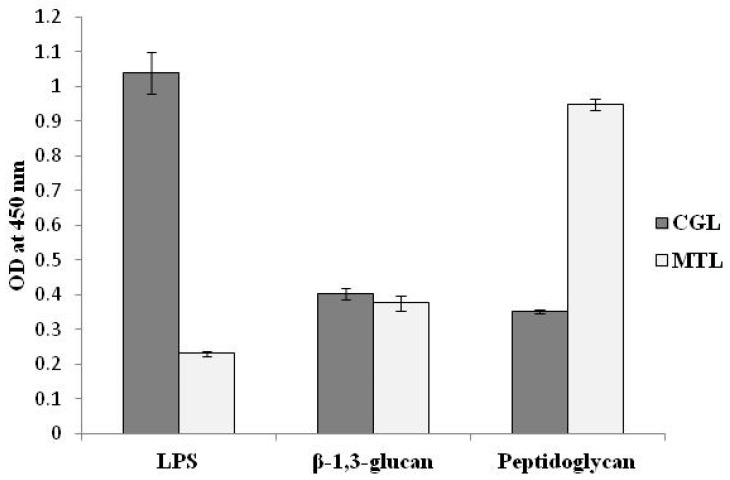
The binding activity of lectins with PAMPs determined by ELLA. Microtiter plates were coated with PAMPs (50 µg/mL, LPS from *Escherichia coli*, β-1,3-glucan from *Euglena gracilis*, PGN from *Staphylococcu aureus*), followed by an incubation with a conjugate of HRP-labeled CGL and MTL at concentrations from 10 to 0.156 mg/mL. The ELLA data are expressed as mean ± SD of three separate experiments.

In our lab the binding activity of lectins with known PAMPs was studied by enzyme-linked lectin assay (ELLA). CGL preferentially bound to lipopolysaccharide (LPS) but had little binding activity toward other examined PAMPs as MTL had a higher affinity with the peptidoglycan (PGN) (unpublished data) (Figure 2). Results of interactions of lectins directly with bacterial cells are summarized in Table 4.

The investigation data have shown the interaction of all lectins with gram-positive and gram-negative bacteria, but in comparison with CGL and MytiLec-1 MTL binds worse and agglutinates *E. coli* as well with less turbidity. At the same time MTL displayed the activity towards *S. aureus*, whereas no obvious growth-suppression effect towards it was observed. In gram-negative bacteria the LPSs are the main glycoconjugates of cell walls [31], in gram-positive bacteria, the lipoteichoic acid [32] and PGN [33]. So LPS and the lipoteichoic acid are the main target glycoconjugates for several lectins and as they are among the most common targets of PRRs can be considered as PAMPs [34]. Both LPS and lipoteichoic acid contain α-galactose or its derivatives as was reported earlier [35,36]. Galactose residues could be target carbohydrates, as they are found in the LPS of different gram-negative bacteria such as *E. coli* and *Vibrio* spp. [31,37]. Perhaps MTL demonstrates less bacteriostatic activity since it preferably interacts with β-galactose.

Many studies have reported that Gal-specific lectins are involved in many aspects of the immune response. For example, the expression level of galectin HrGal from the red abalone *Haliotis rufescens*, was increased at 3 h after infection with *Vibrio anguillarum*, indicating the activation of defense against the pathogen invasion. The decrease of HrGal transcript levels in hemocytes 6–12 h post-challenge may be due to the HrGal translation process in response to a bacterial infection. However, an increase in expression from 24 to 32 h indicates a restoration of the immune response [38]. Similar data were obtained in the clam *Tegillarca granosa* after *Vibrio parahaemolyticus* exposition [39], as well as in the mollusk *Ruditapes philippinarum*, where an expression peak was observed at 24 h after infection with *Vibrio alginolyticus* [40]. The temporary change of CGL and MTL levels were also observed after *Pichia pastoris* challenge. In the mantle, an increase in CGL level was observed, which reached a maximum of two times at 12 h after infection compared with the control group, and then decreased to its initial level after 24 h [41]. Compared with the control group, the levels of MTL expression in the infected group increased and reached the maximum at 24 h (about 1.5 times) after injection, and then decreased to initial values after 48 h [11]. These results suggest that MTL has slightly different cell-binding properties compared with CGL.

The difference in antifungal activity was also observed. Binding of lectins with fungi associated with the mussels was studied quantitatively by ELLA. It was shown that CGL and MTL are active against all fungal species, but the extent of fungal germination inhibition by CGL was impaired slightly (25–65%) compared to the inhibitory activity of MTL (more than 80%) [11,41]. This is probably due to the fact that MTL in the experiment on the direct binding of lectins to PAMPs, interacted most poorly with LPS, and best of all with PGN, which presented in the cell walls of most fungi.

The ability of lectins to inhibit fungal growth varies among fungal species. Changes in the fungal strain susceptibility to inhibition by lectins may reflect differences in the molecular structure of the wall of the fungus and/or be related with the small size of many lectins, which let them penetrate the fungal cell wall [42]. Inhibition of the growth of fungi can occur through the binding of lectin to hyphae, which leads to poor absorption of nutrients, as well as interference in the process of spore germination. Most of the isolated species of the genera Aspergillus, Penicillium, and others were pathogenic and toxigenic fungi [43]. The approach to the study of glycome to determine the structure of glycans on the surface of fungi will give us a lot of useful information for the prevention of mussel disease using lectins.

Although the potential mechanism of the antibacterial and antifungal activity of lectins still needs to be clarified, the observed interaction of MTL and CGL with both bacteria and fungi was significantly inhibited by galactose, a specific sugar ligand, which suggested that the interaction of lectins occurred through the carbohydrate-binding domain. Probably this phenomenon is mediated by the recognition of the above-mentioned PAMP by the mytilectin family members acting as PRRs. Based on data collected to date, it seems that mytilectins can function either directly as inhibitors of bacterial growth or, after agglutination, as modulators of the immune response, causing the action of other molecules and immune cells in mussels.

## 5. Immunomodulatory Activity

Lectins are able to control the immune system through innate immune response leading to recognition and endocytosis of pathogens, and adaptive immune response such as activation of B and T cells and apoptosis. Innate immunity is characterized by non-specific defense through the skin, bone marrow, mucous tissue, or inflammatory components (defensins and cytokines) [44]. Regarding cytokines in invertebrates, some authors report the presence of cytokine-like molecules in mollusks, insects, annelids, echinoderms, and tunicates. Along with morphological data, functional experiments also showed the presence of invertebrate cytokines, which are homologs to those mammals [45].

Marine lectins are structurally diverse, and their unique structures allow them to be potentially used in biomedical applications. SAL, catfish (*Silurus asotus*) egg rhamnose-binding lectin, induced TNF-α expression but not IFN-γ, IL-1β, and IL-10 in Raji cells. The effect was abolished by the addition of specific melibiose but not by sucrose as a negative control. Therefore, SAL-induced cytostatic effect on Raji cells might be partially caused by the TNF-α mediated signaling pathway [46]. GalNAc-specific lectin CEL-I from the marine invertebrate *Cucumaria echinata* is capable of inducing increased secretion of TNF-α and G-CSF by RAW264.7 mouse macrophage cell line in a dose-dependent manner [47]. Furthermore, the activity of CEL-I was much higher than that of PHA-L, a well-known cytokine-inducing lectin [48]. There is limited data on the immunomodulatory activity of marine bivalve lectins compared with those of plant lectins.

**Figure 3:**
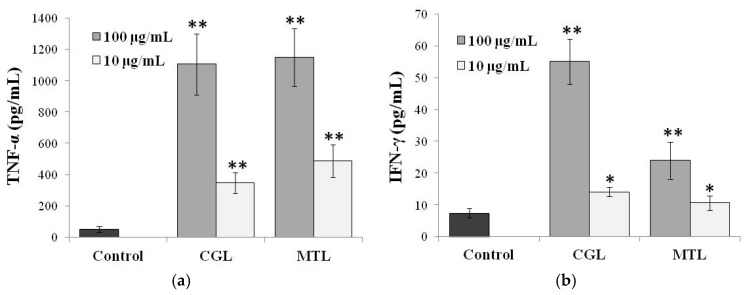
Cytokines-stimulation activity of CGL (unpublished data) and MTL [21] on spontaneous production of (**a**) TNF-α and (**b**) IFN-γ by HPBC. Cells were incubated for 24 h with or without lectin. The levels of cytokines in the culture medium were measured by sandwich enzyme-linked immunosorbent assay (ELISA). The ELISA data are expressed as mean ± SD of three separate experiments. * and ** indicate a significant difference at the level of *p* < 0.05 and *p* < 0.001, respectively, compared to control cells.

The effect of CGL and MTL on spontaneous production of IFN-γ and TNF-α by human peripheral blood cells (HPBC) was studied in our lab (Figure 3). Both lectins activated secretion of TNF-α on a comparable level. But the activity of MTL was much lower with respect to activation of IFN-γ than that of CGL. Perhaps the time before the initiation of cytokine secretion and the lag time of IFN-γ were longer than that of TNF-α, suggesting that the pathways of these cytokines leading to the eventual secretion may be somehow different. Besides, probably, the actual binding sites on the cell surface might have fairly more complex oligosaccharide structure with α-Gal on the end of chains that is recognized by CGL with much higher affinity to Gal. This speculation may also explain the reason for the results that the superior binding ability of CGL to cells may partly account for the higher cytokine-inducing activity of CGL, even though both lectins are Gal-specific lectins. Although the exact secretion mechanisms of these cytokines are still unclear now, it is considered that CGL and MTL are interesting lectins capable of inducing secretion of multiple cytokines by cells.

It is known that the contents of lectins are up-regulated by pathogenic stimulation [11,41]. Increased level of lectins leads to up-regulation of cytokine synthesis, thus providing a synergistic effect. Since the presence of cytokine-like molecules that are homologues to those in mammals has been proven in mollusks, the joint participation of these important messengers in the native immunity of shellfish provides powerful protection against foreign invasion [45]. Although it is unclear now whether or not this is the case for CGL and MTL, we cannot completely rule out the possibility that there are still unknown functional sites on lectin molecules apart from carbohydrate-recognition sites involved in the cytokine-inducing activity. Further detailed study for the structure–activity relationship of CGL and MTL may provide an answer to this question.

**Figure 4:**
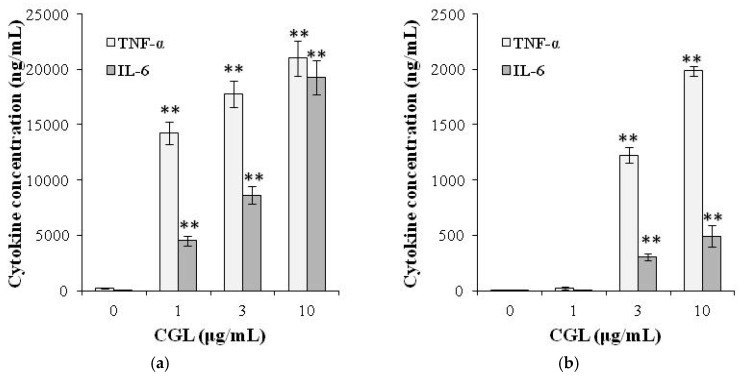
Production of cytokines induced by CGL in (**a**) mouse RAW264.7 and (**b**) human THP-1 macrophages. Cells were incubated for 24 h with or without lectin. The levels of cytokines in the culture medium were measured by ELISA. The ELISA data are expressed as mean ± SD of three separate experiments. ** indicates a significant difference at the level of *p* < 0.001 compared to control cells. Figure was modified from [49].

CGL also induced the production of TNF-α and IL-6 in the RAW264.7 mouse macrophage cell line (Figure 4a) and in the human macrophage cell line THP-1 (Figure 4b). CGL also induced cytokine production in primary cells: macrophages derived from mouse bone marrow, mononuclear cells in human peripheral blood, and macrophages derived from human blood monocytes [49].

As was mentioned above, CGL is specific for N-acetyl-D-galactosamine and galactose. To understand whether TNF-α production mediated by CGL was due to protein–carbohydrate interaction, lectin was incubated with sugars before adding to macrophages. No effect was observed on TNF-α production, suggesting that cytokine production induced by CGL was independent of its carbohydrate binding properties [49].

Further it was shown that CGL not only induces cytokine expression, but improves the bactericidal activity of macrophages [49]. The bactericidal activity of macrophages is characterized by increased phagocytosis and bacterial death. Colony-forming units (CFU) analysis was used to demonstrate that pre-treatment with CGL improved phagocytosis of *E. coli* by macrophages compared to control after 1 h incubation, although statistical significance was not observed (Table 5). These results showed that pre-treatment with CGL of macrophages slightly enhanced phagocytosis of bacteria. However, these results may also indicate a decrease in killing of bacteria. Thus, CFU after a 24-h infection was measured and we found that the number of CFUs in the CGL-pretreated and control cells was 448 and 724, respectively (Table 5). This indicated that approximately 1536 and 956 bacteria (subtracting 24 h CFU from 1 h CFU) were killed in pretreated CGL and control cells, respectively, within 24 h (Table 5). According to obtained data we concluded that CGL slightly enhanced bactericidal activity of macrophages.

CGL induced the production of reactive oxygen species (ROS), which suggests that increased ROS may be partially responsible for increased bactericidal activity, since ROS generated by activated macrophages is an important bactericidal component against intracellular bacteria [50]. Although mitochondrial ROS generated by activated macrophages can also contribute to the bactericidal activity of macrophages [51], the effect of CGL on mitochondrial ROS production is for further study.

**Figure 5:**
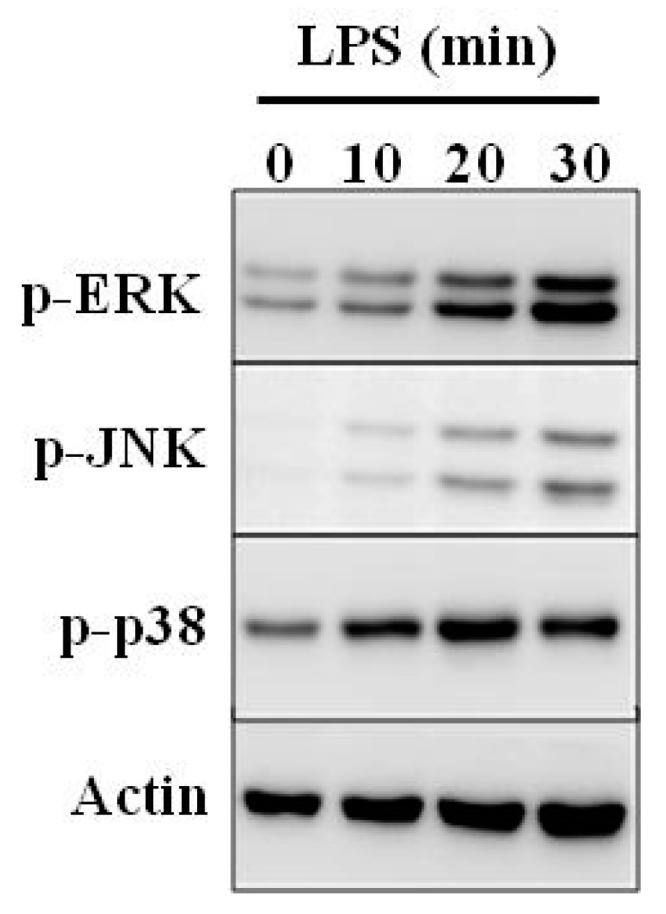
Mechanism of endotoxin tolerance induced by CGL. RAW264.7 macrophages treated with or without CGL (10 µg/mL) for 24 h were subsequently incubated for 0–30 min with or without LPS (1 µg/mL). Figure was modified from [49].

CGL exhibits immunomodulation properties because it not only induces cytokine production, but also induces endotoxin tolerance. Endotoxin tolerance describes a phenomenon when primary treatment of cells with LPS leads to a decrease in sensitivity to a second LPS challenge. Endotoxin tolerance is accompanied by a global decrease in the expression of inflammatory genes [52]. Induction of endotoxin tolerance increases bacterial clearance and improves survival in mice with sepsis [53]. It was found that preliminary incubation of macrophages with CGL led to the phenomenon of endotoxin tolerance. In addition, it also obviously decreased the response to LPS stimulus by reducing secretion of IL-6, generation of NO, expression of iNOS and COX-2. However, this did not affect the secretion of TNF-α [49]. CGL-induced endotoxin tolerance might be due to decreased expression of IRAK2, an important signaling molecule that is important in pathway of NF-κB activation mediated by TLR [53]. Pre-treatment of macrophages with CGL also reduced JNK1/2 phosphorylation, but not ERK1/2 and p38 induced by LPS (Figure 5). Furthermore, pre-treatment with CGL reduced the activation level of NF-κB induced by LPS [49].

Thus, these results suggest that CGL has the potential to be used as an immune modulation agent. One of the interesting findings is that pre-treatment with CGL also leads to the degradation of IRAK2, a downstream TLR4 signaling molecule [49]. In spite of this, pre-treatment with CGL did not inhibit all inflammatory responses mediated by LPS, since it did not reduce expression of TNF-α [49]. It will be interesting to understand the mechanism of TNF-α expression induced by LPS without IRAK2.

## 6. Effect on Tumor Cells

**Figure 6:**
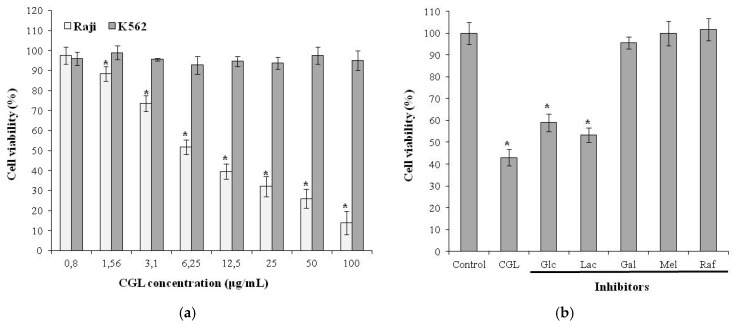
(**a**) Cytotoxic effect of CGL on Raji and K562 cells. (**b**) Inhibition of cytotoxic effect of CGL on Raji cells by addition of various saccharides: Control—only cells with culture medium; CGL—lectin (10 µg/mL) with cells, no inhibitor; Glc—D-glucose; Mel – melibiose; Raf—raffinose; Gal—D-galactose; Lac—lactose. The data are expressed as mean ± SD of three separate experiments. * indicates a significant difference at the level of *p* < 0.05. Figure was modified from [27].

CGL has a very high affinity for the mucin-type glycoproteins as was shown by inhibition of hemagglutination with various glycoproteins [10,54]. These glycoproteins are characterized by a high content of O-glycoside chains densely located on the protein core. Many cellular receptors are known to be mucin-type glycoproteins. In view of the finding that CGL has affinity to (Galα1-4Galβ1-4GlcNAc) motif which is very similar to α-galactosides Gb3 structure (Galα1-4Galβ1-4Glc), expressed on the membrane of some tumor cells including Burkitt‘s lymphoma Raji cells [55], we demonstrated that CGL can recognize Gb3 on the surface of Raji cells leading to dose-dependent cytotoxic effect (Figure 6a) [27]. K562 cells used as negative control were not affected by the addition of CGL. The TF antigen (Galβ1-3GalNAc), expressed on the erythroleukemia K562 cell membrane [56] was not significant for the effect of CGL.

The carbohydrate-dependent mechanism of CGL effect was confirmed by inhibition assay with different glycans. Glucose and β-galactoside lactose as non-specific sugars had no blocking effect (Figure 6b). On the other hand, galactose, α-galactosides raffinose, and melibiose inhibited CGL effect (Figure 6b). These data indicate that α-galactoside structures on cells were critical for the CGL cytotoxic effect.

CGL induced about 30% cell death of MCF7 human breast cancer cells [19]. In contrast, MTL inhibited the viability of MCF7 cells more than 50%. MCF7 cells display not only the Gb3 but also the TF- [57] and Tn-antigens (GalNAc) [58] on the surface of tumor cells confirming the results that CGL and MTL bind to glycans very differently.

MytiLec-1 showed a dose-dependent cytotoxic effect on human Burkitt’s lymphoma Raji [12] and Ramos [59] cells (which have high surface expression of Gb3) but had no such effect on K562 cells. A constructed monomeric mutant has lost all cytotoxic activity against Raji cells. The three sugar binding sites of a single monomer appear to be insufficient for the protein to kill the appropriate target cell lines, and the quaternary structure of the protein also plays a role in its cytotoxicity [59].

Further study of CGL and MytiLec-1 showed that the anti-proliferative effect was due to perforation and inversion of cell membrane. The loss of plasma membrane integrity and exposition of phosphatidylserine at the cell surface are main characteristics of apoptosis, which was confirmed by flow cytometry analysis [27].

**Figure 7:**
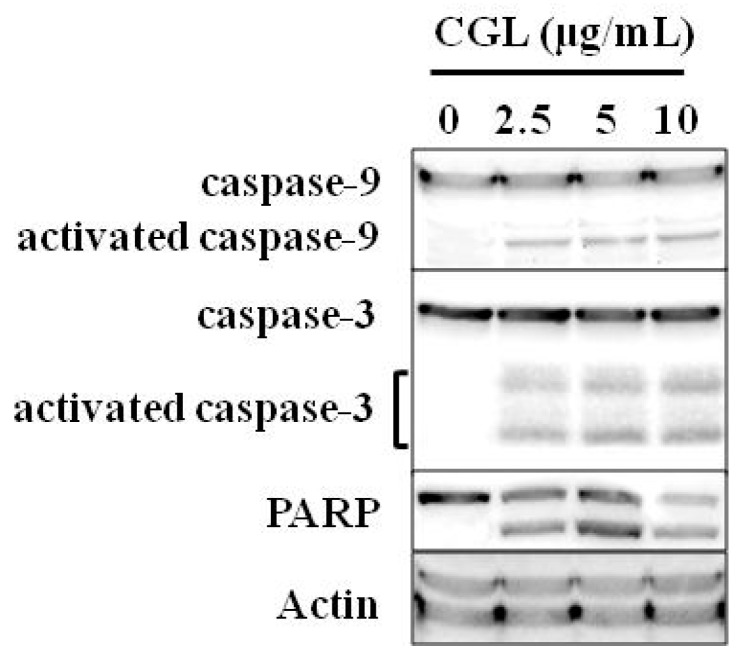
Cell signaling in Raji cells treated with CGL shown by western blotting. Figure was modified from [27].

In addition to the membrane damage, CGL activated caspases cascade which is the most common and important characteristic of apoptosis. Cascade can be triggered by several groups of effectors (caspases-3, -7, -6) and initiators (caspases-8, -9) [60,61]. The initiatory caspases can be activated by different ways. For example, initiation of caspase-9 is associated with intrinsic mitochondrial-dependent pathway [62]. CGL induced apoptosis through caspase pathway as caspase-3, -9, and PARP (poly (ADP-ribose) polymerase) activation was observed (Figure 7). The caspase-9 activation presumably indicates the intrinsic mitochondrial pathway of CGL-induced apoptosis of Raji cells. MytiLec-1 was shown to activate MEK/ERK kinase and stress-dependent kinases JNK and p38. Afterwards, inhibitory protein p21 and TNF-α were expressed, leading to caspase-9/3 activation and apoptosis induction [59].

Gb3 is a cell surface marker found in several different cancer cell lines, suggesting that MytiLec-1 and CGL may be a useful basis for developing new diagnostic agents or treatments for various types of cancer.

## 7. Conclusions

Taken together, members of the mytilectin family could function as an important PRR involved in the antibacterial and antifungal immunity in mollusks through recognizing carbohydrates on the surface of the pathogen. CGL, MTL, and MytiLec-1 may prove a useful basis for the development of new diagnostic agents or treatments for a variety of cancer types. Further study of immune modulation activity is needed.

## Figures and Tables

**Table 1:** Properties of CGL, MTL, and MytiLec-1.

Lectin	MW (Da)	Thermal Stability	pH Dependence	Metal Ion Dependence	Localization in Mussel Tissue	Reference
CGL	16,953	Stable until 50 °C	8–10	Not dependent	Mantle	[10,15]
MTL	16,492	Stable until 50 °C	9–10	Not dependent	Mantle	[11,21]
MytiLec-1	16,812	N.D. ^1^	N.D.	Not dependent	Mantle	[12]

^1^ N.D. means no data.

**Table 2:** Carbohydrate specificity of CGL, MTL, and MytiLec-1.

Carbohydrate	Minimum Inhibitory Concentration, mM		
	CGL [10]	MTL [21]	MytiLec-1 [12]
*N*-Acetyl-d-galactosamine	1.4	0.7	1.6
*N*-Acetyl-d-glucosamine	No inhibition at 80	>50	No inhibition at 100
d-Galactose	5.4	1.7	3.1
d-Glucose	No inhibition at 80	>50	>50
d-Fucose	No inhibition at 80	>50	>50
d-Talose	5.4	N.D. ^1^	25
Lactose	No inhibition at 80	29.2	50
Melibiose	2.0	N.D.	1.6
Raffinose	1.8	N.D.	N.D.
**Glycoproteins**	**Minimum Inhibitory Concentration, mg/mL**		
BSM	0.007	0.0156	No inhibition at 2
Asialo-BSM	0.0017	N.D.	0.2
Fetuin	2	0.0156	No inhibition at 2
Asialofetuin	0.03	0.0156	0.2

^1^ N.D. means no data.

**Table 3:** The most active ligands from the CGL and MTL microarray experiment.

Glycan #	Compound	Fluorescence (%) ^1^	
		CGL [27]	MTL ^2^
559	Galα1-3Galβ1-4GlcNAcβ1-2Mana1-6(Galα1-3Galβ1-4GlcNAcβ1-2Manα1-3)Manβ1-4GlcNAcβ1-4GlcNAc-Sp24	100	87.7
587	Galβ1-4GlcNAcβ1-3Galβ1-4GlcNAcβ1-3Galβ1-4GlcNAcβ1-3Galβ1-4GlcNAcβ1-3Galβ1-4GlcNAcβ1-6(Galβ1-4GlcNAcβ1-3Galβ1-4GlcNAcβ1-3Galβ1-4GlcNAcβ1-3Galβ1-4GlcNAcβ1-3Galβ1-4GlcNAcβ1-2)Manα1-6(Galβ1-4GlcNAcβ1-3Galβ1-4GlcNAcβ1-3Galβ1-4GlcNAcβ1-3Galβ1-4GlcNAcβ1-3Galβ1-4GlcNAcβ1-2Manα1-3)Manβ1-4GlcNAcβ1-4(Fucα1-6)GlcNAcβ-Sp24	98.7	100
362	Galα1-3Galβ1-4GlcNAcβ1-2Manα1-6(Galα1-3Galβ1-4GlcNAcβ1-2Manα1-3)Manβ1-4GlcNAcβ1-4GlcNAcβ-Sp20	96.4	91.5
402	Galα1-4Galβ1-3GlcNAcβ1-2Manα1-6(Galα1-4Galβ1-3GlcNAcβ1-2Manα1-3)Manβ1-4GlcNAcβ1-4GlcNAcβ-Sp19	78.2	72.5
583	Galβ1-4GlcNAcβ1-3Galβ1-4GlcNAcβ1-3Galβ1-4GlcNAcβ1-3Galβ1-4GlcNAcβ1-6(Galβ1-4GlcNAcβ1-3Galβ1-4GlcNAcβ1-3Galβ1-4GlcNAcβ1-3Galβ1-4GlcNAcβ1-2)Manα1-6(Galβ1-4GlcNAcβ1-3Galβ1-4GlcNAcβ1-3Galβ1-4GlcNAcβ1-3Galβ1-4GlcNAcβ1-2Manα1-3)Manβ1-4GlcNAcβ1-4(Fucα1-6)GlcNAcβ-Sp24	66.5	72.4
122	Galα1-4Galβ1-4Glcβ-Sp0	24.5	23.3
72	Fucα1-2Galβ1-4(Fucα1-3)GlcNAcβ-Sp8	0.15	0.03

^1^ Expressed as a percentage of the ligand with strongest fluorescence. ^2^ Unpublished data.

**Table 4:** Antimicrobial and antifungal activity of CGL [15], MTL [11], and MytiLec-1 [14].

Bacteria	Binding Activity ^1^		Agglutination ^2^			Growth Suppressive Activity (%)		
	CGL	MTL	CGL	MTL	MytiLec-1	CGL	MTL	MytiLec-1
*Candida albicans*	0.65 ± 0.01	0.44 ± 0.05	++	+++	N.D. ^3^	- ^4^	-	N.D.
*Vibrio proteolyticus*	1.42 ± 0.04	0.37 ± 0.14	+++	++	N.D.	39.9 ± 5	-	N.D.
*Escherichia coli*	1.63 ± 0.09	0.48 ± 0.06	++	+	++	46 ± 5	10.6 ± 7	58 ± 5
*Bacillus subtilis*	0.74 ± 0.07	0.26 ± 0.07	+++	++	++	85 ± 8	62 ± 6	74 ± 8
*Staphylococcus aureus*	0.39 ± 0.06	0.49 ± 0.01	++	++	+	68 ± 6	-	61 ± 36

^1^ Binding intensity determined by ELLA and measured at 450 nm; ^2^ Strong (+++), good (++), and weak (+) binding or agglutination, respectively; ^3^ Not determined; ^4^ Absence (-) of the effect.

**Table 5:** Influence of CGL on RAW264.7 macrophage bactericidal activity (values were calculated using data from [49]).

CGL (μg/mL)	CFU (×10^6^)		Killed Bacteria (×10^6^)
	1 h after Infection	24 h after Infection	
**0**	1680 ± 370	724 ± 196	956
10	1984 ± 792	448 ± 166	1536

## References

[1] Sharon N., Lis H. (2007). Lectins.

[2] McGreal E., Martinez-Pomares L., Gordon S. (2004). Divergent roles for C-type lectins expressed by cells of the innate immune system. Mol. Immunol..

[3] Sharon N. (2007). Lectins: Carbohydrate-specific Reagents and Biological Recognition Molecules. J. Biol. Chem..

[4] Liu Z., Luo Y., Zhou T.-T., Zhang W.-Z. (2013). Could plant lectins become promising anti-tumour drugs for causing autophagic cell death?. Cell Prolif..

[5] Cardoso M.R.D., Mota C.M., Ribeiro D.P., Noleto P.G., Andrade W.B.F., Souza M.A., Silva N.M., Mineo T.W.P., Mineo J.R., Silva D.A.O. (2012). Adjuvant and immunostimulatory effects of a D-galactose-binding lectin from Synadenium carinatum latex (ScLL) in the mouse model of vaccination against neosporosis. Vet. Res..

[6] Wang J.-H., Kong J., Li W., Molchanova V., Chikalovets I., Belogortseva N., Luk’yanov P., Zheng Y.-T. (2006). A beta-galactose-specific lectin isolated from the marine worm Chaetopterus variopedatus possesses anti-HIV-1 activity. Comp. Biochem. Physiol. C Toxicol. Pharmacol..

[7] Allen H.J., Kisailus E.C. (1992). Glycoconjugates: Composition, Structure, and Function.

[8] Chatterjee B.P., Adhya M. (2013). Lectins with Varying Specificity and Biological Activity from Marine Bivalves. Marine Proteins and Peptides.

[9] Adhya M., Singha B. (2016). Gal/GalNAc specific multiple lectins in marine bivalve Anadara granosa. Fish Shellfish Immunol..

[10] Belogortseva N.I., Molchanova V.I., Kurika A.V., Skobun A.S., Glazkova V.E. (1998). Isolation and characterization of new GalNAc/Gal-specific lectin from the sea mussel Crenomytilus grayanus. Comp. Biochem. Physiol. C Pharmacol. Toxicol. Endocrinol..

[11] Chikalovets I.V., Kovalchuk S.N., Litovchenko A.P., Molchanova V.I., Pivkin M.V., Chernikov O.V. (2016). A new Gal/GalNAc-specific lectin from the mussel Mytilus trossulus: Structure, tissue specificity, antimicrobial and antifungal activity. Fish Shellfish Immunol..

[12] Fujii Y., Dohmae N., Takio K., Kawsar S.M., Matsumoto R., Hasan I., Koide Y., Kanaly R.A., Yasumitsu H., Ogawa Y. (2012). A lectin from the mussel Mytilus galloprovincialis has a highly novel primary structure and induces glycan-mediated cytotoxicity of globotriaosylceramide-expressing lymphoma cells. J. Biol. Chem..

[13] Chikalovets I.V., Mizgina T.O., Molchanova V.I., Ovcharenko Y.S., Chernikov O.V. (2017). Isolation and Characterization of Lectin from the Scallop Patinopecten yessoensis. Chem. Nat. Compd..

[14] Hasan I., Gerdol M., Fujii Y., Rajia S., Koide Y., Yamamoto D., Kawsar S.M.A., Ozeki Y. (2016). CDNA and Gene Structure of MytiLec-1, A Bacteriostatic R-Type Lectin from the Mediterranean Mussel (Mytilus galloprovincialis). Mar. Drugs.

[15] Kovalchuk S.N., Chikalovets I.V., Chernikov O.V., Molchanova V.I., Li W., Rasskazov V.A., Lukyanov P.A. (2013). CDNA cloning and structural characterization of a lectin from the mussel Crenomytilus grayanus with a unique amino acid sequence and antibacterial activity. Fish Shellfish Immunol..

[16] Finn R.D., Coggill P., Eberhardt R.Y., Eddy S.R., Mistry J., Mitchell A.L., Potter S.C., Punta M., Qureshi M., Sangrador-Vegas A. (2016). The Pfam protein families database: Towards a more sustainable future. Nucleic Acids Res..

[17] Fujii Y., Gerdol M., Hasan I., Koide Y., Matsuzaki R., Ikeda M., Rajia S., Ogawa Y., Kawsar S.M.A., Ozeki Y. (2018). Phylogeny and Properties of a Novel Lectin Family with β-Trefoil Folding in Mussels. Trends Glycosci. Glycotechnol..

[18] Terada D., Kawai F., Noguchi H., Unzai S., Hasan I., Fujii Y., Park S.-Y., Ozeki Y., Tame J.R.H. (2016). Crystal structure of MytiLec, a galactose-binding lectin from the mussel Mytilus galloprovincialis with cytotoxicity against certain cancer cell types. Sci. Rep..

[19] Liao J.H., Chien C.T., Wu H.Y., Huang K.F., Wang I., Ho M.R., Tu I.F., Lee I.M., Li W., Shih Y.L. (2016). A Multivalent Marine Lectin from Crenomytilus grayanus Possesses Anti-cancer Activity through Recognizing Globotriose Gb3. J. Am. Chem. Soc..

[20] Gerdol M., Venier P. (2015). An updated molecular basis for mussel immunity. Fish Shellfish Immunol..

[21] Chikalovets I.V., Kondrashina A.S., Chernikov O.V., Molchanova V.I., Luk’yanov P.A. (2013). Isolation and general characteristics of lectin from the mussel Mytilus trossulus. Chem. Nat. Compd..

[22] Chichvarkhin A.I., Kartavtsev I.F., Kafanov A.I. (2000). Genetic connections between some species of Mytilidae (Mollusca: Bivalvia) from the northern part of the Pacific Ocean. Genetika.

[23] Houser J., Komárek J., Kostlánová N., Cioci G., Imberty A., Wimmerová M. (2012). Protein oligomerization in Aleuria aurantia lectin family—Importance and difficulties. Mater. Struct. Chem. Biol. Phys. Technol..

[24] Chikalovets I.V., Molchanova V.I., Chernikov O.V., Luk’Yanov P.A. (2014). Domain organization of lectin from the mussel Crenomytilus grayanus. Chem. Nat. Compd..

[25] Cho M., Cummings R.D. (1995). Galectin-1, a beta-galactoside-binding lectin in Chinese hamster ovary cells. I. Physical and chemical characterization. J. Biol. Chem..

[26] Terada D., Voet A.R.D., Noguchi H., Kamata K., Ohki M., Addy C., Fujii Y., Yamamoto D., Ozeki Y., Tame J.R.H. (2017). Computational design of a symmetrical β-trefoil lectin with cancer cell binding activity. Sci. Rep..

[27] Chernikov O., Kuzmich A., Chikalovets I., Molchanova V., Hua K.-F. (2017). Lectin CGL from the sea mussel Crenomytilus grayanus induces Burkitt’s lymphoma cells death via interaction with surface glycan. Int. J. Biol. Macromol..

[28] Oyelaran O., Gildersleeve J.C. (2009). Glycan arrays: Recent advances and future challenges. Curr. Opin. Chem. Biol..

[29] Cheung R.C., Wong J.H., Pan W., Chan Y.S., Yin C., Dan X., Ng T.B. (2015). Marine lectins and their medicinal applications. Appl. Microbiol. Biotechnol..

[30] Toubiana M., Gerdol M., Rosani U., Pallavicini A., Venier P., Roch P. (2013). Toll-like receptors and MyD88 adaptors in Mytilus: Complete cds and gene expression levels. Dev. Comp. Immunol..

[31] Kubler-Kielb J., Lai W.-T., Schneerson R., Vinogradov E. (2011). The structure of the Escherichia coli O148 lipopolysaccharide core region and its linkage to the O-specific polysaccharide. Carbohydr. Res..

[32] Poxton I.R. (2015). Teichoic Acids, Lipoteichoic Acids and Other Secondary Cell Wall and Membrane Polysaccharides of Gram-Positive Bacteria. Molecular Medical Microbiology.

[33] Vollmer W. (2015). Peptidoglycan. Molecular Medical Microbiology.

[34] Gaudet R.G., Sintsova A., Buckwalter C.M., Leung N., Cochrane A., Li J., Cox A.D., Moffat J., Gray-Owen S.D. (2015). Cytosolic detection of the bacterial metabolite HBP activates TIFA-dependent innate immunity. Science.

[35] Wollin R., Creeger E.S., Rothfield L.I., Stocker B.A., Lindberg A.A. (1983). Salmonella typhimurium mutants defective in UDP-D-galactose:lipopolysaccharide alpha 1,6-D-galactosyltransferase. Structural, immunochemical, and enzymologic studies of rfaB mutants. J. Biol. Chem..

[36] Klein R.A., Hartmann R., Egge H., Behr T., Fischer W. (1996). The aqueous solution structure of a lipoteichoic acid from Streptococcus pneumoniae strain R6 containing 2,4-diamino-2,4,6-trideoxy-galactose: Evidence for conformational mobility of the galactopyranose ring. Carbohydr. Res..

[37] Valiente E., Jiménez N., Merino S., Tomás J.M., Amaro C. (2008). Vibrio vulnificus biotype 2 serovar E gne but not galE is essential for lipopolysaccharide biosynthesis and virulence. Infect. Immun..

[38] Maldonado-Aguayo W., Teneb J., Gallardo-Escárate C. (2014). A galectin with quadruple-domain from red abalone Haliotis rufescens involved in the immune innate response against to Vibrio anguillarum. Fish Shellfish Immunol..

[39] Bao Y., Shen H., Zhou H., Dong Y., Lin Z. (2013). A tandem-repeat galectin from blood clam Tegillarca granosa and its induced mRNA expression response against bacterial challenge. Genes Genom..

[40] Moreira R., Balseiro P., Romero A., Dios S., Posada D., Novoa B., Figueras A. (2012). Gene expression analysis of clams Ruditapes philippinarum and Ruditapes decussatus following bacterial infection yields molecular insights into pathogen resistance and immunity. Dev. Comp. Immunol..

[41] Chikalovets I.V., Chernikov O.V., Pivkin M.V., Molchanova V.I., Litovchenko A.P., Li W., Lukyanov P.A. (2015). A lectin with antifungal activity from the mussel Crenomytilus grayanus. Fish Shellfish Immunol..

[42] Van Parijs J., Joosen H.M., Peumans W.J., Geuns J.M., Van Laere A.J. (1992). Effect of the Urtica dioica agglutinin on germination and cell wall formation of Phycomyces blakesleeanus Burgeff. Arch. Microbiol..

[43] Zvereva L.V., Vysotskaya M.A. (2005). Filamentous Fungi Associated with Bivalve Mollusks from Polluted Biotopes of Ussuriiskii Bay, Sea of Japan. Russ. J. Mar. Biol..

[44] Kang H.K., Lee H.H., Seo C.H., Park Y. (2019). Antimicrobial and immunomodulatory properties and applications of marine-derived proteins and peptides. Mar. Drugs.

[45] Malagoli D., Sacchi S., Ottaviani E. (2010). Lectins and cytokines in celomatic invertebrates: Two tales with the same end. Invertebr. Surviv. J..

[46] Hosono M., Sugawara S., Matsuda A., Tatsuta T., Koide Y., Hasan I., Ozeki Y., Nitta K. (2014). Binding profiles and cytokine-inducing effects of fish rhamnose-binding lectins on Burkitt’s lymphoma Raji cells. Fish Physiol. Biochem..

[47] Yamanishi T., Yamamoto Y., Hatakeyama T., Yamaguchi K., Oda T. (2007). CEL-I, an invertebrate N-acetylgalactosamine-specific C-type lectin, induces TNF-alpha and G-CSF production by mouse macrophage cell line RAW264.7 cells. J. Biochem..

[48] Chang S.H., Mun S.H., Ko N.Y., Lee J.H., Jun M.H., Seo J.Y., Kim Y.M., Choi W.S., Her E. (2005). The synergistic effect of phytohemagglutinin and interferon-gamma on the expression of tumor necrosis factor-alpha from RAW 264.7 cells. Immunol. Lett..

[49] Chernikov O.V., Wong W.-T., Li L.-H., Chikalovets I.V., Molchanova V.I., Wu S.-H., Liao J.-H., Hua K.-F. (2017). A GalNAc/Gal-specific lectin from the sea mussel Crenomytilus grayanus modulates immune response in macrophages and in mice. Sci. Rep..

[50] Lambeth J.D. (2004). NOX enzymes and the biology of reactive oxygen. Nat. Rev. Immunol..

[51] West A.P., Brodsky I.E., Rahner C., Woo D.K., Erdjument-Bromage H., Tempst P., Walsh M.C., Choi Y., Shadel G.S., Ghosh S. (2011). TLR signalling augments macrophage bactericidal activity through mitochondrial ROS. Nature.

[52] Fan H., Cook J.A. (2004). Molecular mechanisms of endotoxin tolerance. J. Endotoxin Res..

[53] Wheeler D.S., Lahni P.M., Denenberg A.G., Poynter S.E., Wong H.R., Cook J.A., Zingarelli B. (2008). Induction of endotoxin tolerance enhances bacterial clearance and survival in murine polymicrobial sepsis. Shock.

[54] Furtak V.A., Kurika A.V., Belogortseva N.I., Chikalovets I.V., Kleshch Y. (1999). Cell localization of mucin-type receptors assayed with novel GalNac/Gal-specific lectin from sea mussel Crenomytilus grayanus in human colon tumors. Bull. Exp. Biol. Med..

[55] Nudelman E., Kannagi R., Hakomori S., Parsons M., Lipinski M., Wiels J., Fellous M., Tursz T. (1983). A glycolipid antigen associated with Burkitt lymphoma defined by a monoclonal antibody. Science.

[56] Cao Y., Merling A., Karsten U., Goletz S., Punzel M., Kraft R., Butschak G., Schwartz-Albiez R. (2008). Expression of CD175 (Tn), CD175s (sialosyl-Tn) and CD176 (Thomsen-Friedenreich antigen) on malignant human hematopoietic cells. Int. J. Cancer.

[57] Geiger P., Mayer B., Wiest I., Schulze S., Jeschke U., Weissenbacher T. (2016). Binding of galectin-1 to breast cancer cells MCF7 induces apoptosis and inhibition of proliferation in vitro in a 2D- and 3D- cell culture model. BMC Cancer.

[58] Freire T., Bay S., von Mensdorff-Pouilly S., Osinaga E. (2005). Molecular Basis of Incomplete O-Glycan Synthesis in MCF-7 Breast Cancer Cells: Putative Role of MUC6 in Tn Antigen Expression. Cancer Res..

[59] Hasan I., Sugawara S., Fujii Y., Koide Y., Terada D., Iimura N., Fujiwara T., Takahashi K.G., Kojima N., Rajia S. (2015). MytiLec, a Mussel R-Type Lectin, Interacts with Surface Glycan Gb3 on Burkitt’s Lymphoma Cells to Trigger Apoptosis through Multiple Pathways. Mar. Drugs.

[60] Chang H.Y., Yang X. (2000). Proteases for cell suicide: Functions and regulation of caspases. Microbiol. Mol. Biol. Rev..

[61] Stennicke H.R., Salvesen G.S. (1998). Properties of the caspases. Biochim. Biophys. Acta.

[62] Dias N., Bailly C. (2005). Drugs targeting mitochondrial functions to control tumor cell growth. Biochem. Pharmacol..

